# Supplementary material for: Quantitative analysis of nuclear pore complex organization in Schizosaccharomyces pombe
Source: Life Sci Alliance. 2022 Mar 30;5(7):e202201423. doi: 10.26508/lsa.202201423 (PMC8967992; doi:10.26508/lsa.202201423)
Supplement: Supplementary file 1 [file LSA-2022-01423_TableS1.docx]

**Table S1: Summary statistics for Nsp1-GFP NPC analysis**^†^

| Stage | Cell Length (μm) | Nuclear Surface Area (μm^2^) | Nuclear Volume (μm^3^) | Nucleus Sphericity | Number NPCs | NPC Density (NPCs per μm^2^) | Points Removed | N |
| --- | --- | --- | --- | --- | --- | --- | --- | --- |
| Early G2 | 8.7 ± 0.5 | 16.9 ± 2.7 | 5.9 ± 1.4 | 0.93 ± 0.02 | 95.3 ± 19.9 | 5.7 ± 1.0 | 0.8 ± 1.2 | 233 |
| Mid G2 | 10.2 ± 0.4 | 18.4 ± 2.8 | 6.7 ± 1.6 | 0.93 ± 0.02 | 105.1 ± 20.2 | 5.8 ± 1.0 | 0.9 ± 1.5 | 174 |
| Late G2/  Early M | 12.7 ± 1.2 | 21.8 ± 4.1 | 8.7 ± 2.4 | 0.93 ± 0.02 | 123.7 ± 26.6 | 5.8 ± 1.2 | 0.9 ± 1.3 | 317 |
| Late M | 14.0 ± 0.9 | 14.6 ± 2.6 | 4.6 ± 1.3 | 0.91 ± 0.02 | 77.5 ± 13.9 | 5.4 ± 1.1 | 1.2 ± 1.8 | 122 |
| G1/S | 14.2 ± 1.0 | 15.5 ± 2.8 | 5.1 ± 0.5 | 0.92 ± 0.02 | 81.1 ± 17.1 | 5.3 ± 0.9 | 0.9 ± 1.2 | 162 |

^†^*Values not corrected for undercounting*

Summary statistics from four independent biological replicates of Nsp1-GFP 3D-SIM imaging experiments. Stages assigned as described in *Materials and Methods*. Surface Area, Volume, Sphericity and NPC Density values derived from the computed 3D convex hull. Points Removed represents the average number of NPC points removed during complex hull optimization as described in *Materials and Methods*. Values shown were not corrected for undercounting.
