# Supplementary material for: Quantitative analysis of nuclear pore complex organization in Schizosaccharomyces pombe
Source: Life Sci Alliance. 2022 Mar 30;5(7):e202201423. doi: 10.26508/lsa.202201423 (PMC8967992; doi:10.26508/lsa.202201423)
Supplement: Supplementary file 3 [file LSA-2022-01423_TableS2.docx]

**Table S2: Yeast strains**

| **Strain** | **Genotype** | **Figure** | **Source/Reference** |
| --- | --- | --- | --- |
| fySLJ738 | *h?, nsp1-GFP-KanMX6, ppc89-mCherry-NatMX6, his3-D1, leu1-32, ura4-D18, ade6-M210* | Fig. 1A-E; S1E, H; 5B-G; 6B | This study |
| fySLJ035 | *h-, cut11:GFP:ura4+, leu1-32, ura4-D18* | Fig. 1B | J.R. McIntosh, (Mcl316) |
| fySLJ485 | *h-, nup146-GFP-KanMX6, his3-D1, leu1-32, ura4-D18, ade6-M210* | Fig. 1B; S1E; 3B | This study |
| fySLJ745 | *h-, nup60-GFP-KanMX6, ppc89-mCherry-NatMX6, his3-D1, leu1-32, ura4-D18, ade6-M210* | Fig. 1B; S1H; 3B; 5F-G, S3 | This study |
| fySLJ530 | *h?, nup97-GFP-KanMX6, ppc89-mCherry-NatMX6, his3-D1, leu1-32, ura4-D18, ade6-M210* | Fig. 1B; S1E,H; 5F-G | This study |
| fySLJ822 | *h90, nup37-GFP-KanMX6, ppc89-mCherry-NatMX6, his3-D1, leu1-32, ura4-D18, ade6-M210* | Fig. S1E; 5F-G, S3 | This study |
| fySLJ747 | *h+, nup85-GFP-KanMX6, ppc89-mCherry-NatMX6, his3-D1, leu1-32, ura4-D18, ade6-M210* | Fig. S1E,H; 5F-G | This study |
| fySLJ566 | *h?, nup44-GFP-KanMX6, nup40-mCherry-NatMX6, his3-D1, leu1-32, ura4-D18, ade6-M210* | Fig. S1A-C | This study |
| fySLJ714 | *h-, cdc7-GFP-KanMX6, nsp1-mCherry-NatMX6, his3-D1,*  *leu1-32, ura4-D18, ade6-M210* | Fig. S1A | This study |
| fySLJ771 | *h?, cut11:GFP:ura4+, nup37-mCherry-NatMX6, leu1-32, ura4-D18* | Fig. S1D | This study |
| fySLJ772 | *h?, cut11:GFP:ura4+, nup60-mCherry-NatMX6, leu1-32, ura4-D18* | Fig. S1D | This study |
| fySLJ457 | *h+, nsp1-mCherry-NatMX6, his3-D1, leu1-32, ura4-D18, ade6-M210* | Fig. S1E; 2A; S2A; 3 | This study |
| fySLJ944 | *h+, wee1-50, nup37-mCherry-HygMX6, ade6-M210, leu1-32, ura4-D18* | Fig. 2B; S2B | This study |
| fySLJ1112 | *h?, ppc89-mCherry-NatMX6, nsp1-GFP-KanMX6, cdc25.22, leu1-32, ura4-D18* | Fig. 2C; S2C | This study |
| fySLJ541 | *h-, nsp1-mCherry-NatMX6, (prototroph)* | Fig. 2D; S2D | This study |
| fySLJ1135 | *h?, atg1∆::KanMX6, nsp1-mCherry-NatMX6 (prototroph)* | Fig. 2D; S2D | This study |
| fySLJ1136 | *h?, atg8∆::KanMX6, nsp1-mCherry-NatMX6 (prototroph)* | Fig. 2D; S2D | This study |
| fySLJ515 | *h?, nup132∆::KanMX6, nsp1-mCherry-NatMX6, leu1-32, ura4-D18* | Fig. 3A-C | This study |
| fySLJ748 | *h+, nup37-GFP-KanMX6, his3-D1, leu1-32, ura4-D18,*  *ade6-M210* | Fig. 3B | This study |
| fySLJ879 | *h?, nem1∆::KanMX6, nsp1-mCherry-NatMX6, leu1-32, ura4-D18* | Fig. 3D-E; S2E,F | This study |
| fySLJ1147 | *h?, nup132∆::KanMX6, nsp1-mCherry-NatMX6, Sad1-GFP-NatMX6* | Fig. 3F,G; Video S1 | This study |
| fySLJ712 | *h?, nuc1-mCherry-NatMX6, nup44-GFP-KanMX6, his3-D1, leu1-32, ura4-D18, ade6-M210* | Fig. 4A-D | This study |
| fySLJ713 | *h?, nuc1-mCherry-NatMX6, nup146-GFP-KanMX6, his3-D1, leu1-32, ura4-D18, ade6-M210* | Fig. 4B-D | This study |
| fySLJ997 | *h?, nuc1-mCherry-NatMX6, nup124-GFP-KanMX6, his3-D1, leu1-32, ura4-D18, ade6-M210* | Fig. 4B-D | This study |
| fySLJ998 | *h-, nuc1-mCherry-NatMX6, nup211-GFP-KanMX6, his3-D1, leu1-32, ura4-D18, ade6-M210* | Fig. 4B-D | This study |
| fySLJ1001 | *h-, nuc1-mCherry-NatMX6, nup97-mNeonGreen-HygMX6, his3-D1, leu1-32, ura4-D18, ade6-M210* | Fig. 4B-D | This study |
| fySLJ1017 | *h?, nuc1-mCherry-NatMX6, nup60-GFP-KanMX6, his3-D1, leu1-32, ura4-D18, ade6-M210* | Fig. 4B-D | This study |
| fySLJ1020 | *h?, nuc1-mCherry-NatMX6, nup61-GFP-KanMX6, his3-D1, leu1-32, ura4-D18, ade6-M210* | Fig. 4B-D | This study |
| fySLJ1021 | *h?, nuc1-mCherry-NatMX6, alm1-GFP-KanMX6, his3-D1, leu1-32, ura4-D18, ade6-M210* | Fig. 4B-D | This study |
| fySLJ1022 | *h?, nuc1-mCherry-NatMX6, seh1-GFP-KanMX6, his3-D1, leu1-32, ura4-D18, ade6-M210* | Fig. 4B-D | This study |
| fySLJ1211 | *h?, alm1-GFP-KanMX6, nsp1-mCh-NatMX6, his3-D1, leu1-32, ura4-D18, ade6-M210* | Fig. 4E-F | This study |
| fySLJ1208 | *h+, les1-mNeonGreen-HygMX6, ppc89-mCherry-NatMX6, his3-D1, leu1-32, ura4-D18, ade6-M210* | Fig. 5B | This study |
| fySLJ1122 | *h+, sad1-GFP-KanMX6, adh15::mCherry-atb2-NatMX6* | Fig. 5C | This study |
| fySLJ746 | *h+, nup82-GFP-KanMX6, ppc89-mCherry-NatMX6, his3-D1, leu1-32, ura4-D18, ade6-M210* | Fig. 5F-G, S3 | This study |
| fySLJ867 | *h?, alm1-GFP-KanMX6, ppc89-mCherry-NatMX6, his3-D1, leu1-32, ura4-D18, ade6-M210* | Fig. 5F-G | This study |
| fySLJ577 | *h+, lem2-GFP-KanMX6, ppc89-mCherry-NatMX6, his3-D1, leu1-32, ura4-D18, ade6-M210* | Fig. 6A | Bestul et al., 2021 |
| fySLJ845 | *h?, nsp1-GFP-KanMX6, ppc89-mCherry-NatMX6, ima1∆::HygMX6, leu1-32, ura4-D18* | Fig. 6B | This study |
| fySLJ849 | *h?, nsp1-GFP-KanMX6, ppc89-mCherry-NatMX6, lem2∆::HygMX6, leu1-32, ura4-D18* | Fig. 6B | This study |
| fySLJ860 | *h?, nsp1-GFP-KanMX6, ppc89-mCherry-NatMX6, man1∆::HygMX6, leu1-32, ura4-D18* | Fig. 6B | This study |
| fySLJ1209 | *h?, nsp1-GFP-KanMX6, ppc89-mCherry-NatMX6, csi1∆::HygMX6, leu1-32, ura4-D18* | Fig. 6E | This study |
| fySLJ1117 | *h?, nsp1-GFP-KanMX6, ppc89-mCherry-NatMX6, lem2∆::HygMX6, ura4::nmt41-lem2FL-3xHA-ura4+, leu1-32, ura4-D18* | Fig. 6D, S4 | This study |
| fySLJ1142 | *h?, nsp1-GFP-KanMX6, ppc89-mCherry-NatMX6, lem2∆::HygMX6, ura4::nmt41-lem2∆C-3xHA-ura4+, leu1-32, ura4-D18* | Fig. 6D, S4 | This study |
| fySLJ1144 | *h?, nsp1-GFP-KanMX6, ppc89-mCherry-NatMX6, lem2∆::HygMX6, ura4::nmt41-lem2∆N-3xHA-ura4+, leu1-32, ura4-D18* | Fig. 6D, S4 | This study |
